# The Impact of Mindset on Women’s Work-Life Balance and Mental Health: Foundations for Career Success

**DOI:** 10.1192/j.eurpsy.2026.11929

**Published:** 2026-07-31

**Authors:** C. Sarkis

**Affiliations:** Psychology, Pepperdine University, Calabasas, United States

## Abstract

**Introduction:**

This study investigates how women’s mindsets influence their ability to achieve work-life balance and maintain mental health, which are critical factors for sustainable career success. Given the increasing professional demands alongside personal responsibilities, women’s psychological resilience and mindset set the tone for managing these complexities effectively.

**Objectives:**

The primary objective is to explore the relationship between mindset, work-life balance, and mental health among working women. Specifically, the study aims to identify how a positive, growth-oriented mindset correlates with better stress management, boundary setting, and enhanced career outcomes.

**Methods:**

A qualitative approach was utilized, measuring mindset orientation, perceived work-life balance, and mental health indices, with qualitative interviews exploring personal experiences of career challenges and coping mechanisms. A qualitative approach was utilized to assess mindset orientation, perceived work-life balance, and mental health indices. In-depth interviews explored participants’ personal experiences with career challenges and coping mechanisms.

**Results:**

Findings reveal that women who exhibit a growth mindset demonstrate significantly higher work-life balance satisfaction and lower levels of occupational stress and burnout. Qualitative data support these results, illustrating that mindset influences practical behaviors such as prioritizing self-care, seeking social support, and setting professional boundaries. Moreover, these women reported greater career advancement and psychological well-being.

**Conclusions:**

The study concludes that mindset functions as a foundational element shaping women’s experiences balancing work and life demands, directly impacting mental health and career success. Interventions promoting a positive mindset could enhance women’s resilience, leading to improved well-being and professional outcomes. Women with a goal-oriented mindset were less likely to engage in self-defeating attitudes that hinder progress. Future research should develop targeted mindset-training programs to support women navigating complex work-life dynamics.

**Disclosure of Interest:**

None Declared

